# New indices regarding the dominance and diversity of communities, derived from sample variance and standard deviation

**DOI:** 10.1016/j.heliyon.2019.e02606

**Published:** 2019-10-11

**Authors:** Ashwani Kumar Thukral, Renu Bhardwaj, Vinod Kumar, Anket Sharma

**Affiliations:** a Department of Botanical and Environmental Sciences, Guru Nanak Dev University, Amritsar, Punjab 143005, India; b Department of Botany, DAV University, Sarmastpur, Jalandhar, Punjab 144012, India; c State Key Laboratory of Subtropical Silviculture, Zhejiang A & F University, Hangzhou, 311300, China

**Keywords:** Ecology, Environmental chemistry, Environmental geochemistry, Environmental impact assessment, Environmental risk assessment, Environmental toxicology, Diversity indices, Simpson's dominance, Shannon's entropy, Sample variance, Covariance, Binary information plots

## Abstract

Dominance and diversity are important characteristics for the description of communities. The most commonly used indices are Simpson's dominance indexand Shannon's and Simpson's indices of diversity. This paper uses the basic concepts of statistics as applied to community analysis to develop new dominance and diversity indices that will enable scientists to establish correlations among various indices. The present study proves that the variance of the number of individuals of different species in a sample can be used to calculateSimpson's dominance and diversity indices. New indices have been developed from the ratios ofthe variance to number of species, and the mean number of individuals per species in a quadrat. A wide range of data, varying from high dominance to high evenness, was simulated for 25 quadrats, with each quadrat having ten species and 100 individuals in different combinations. Variance and standard deviation-based indices were computed using the simulated data and were found to be highly correlated with Simpson's and Shannon's indices. The proposed indices will give both the dominance and diversity of a community on the same scale based on the same statistic. Another important contribution of the present study relates to the variance of a sample consisting of a single value. It has been proved that the variance of a sample having only one value is equal to the square of that value. The paper establishes a new link between diversity studies and statistics.

## 1 Introduction

The computation of diversity indices is a key tool for the quantitative characterisation of community statistics [1]. These indices help in the appraisalof the ecological and biological features of the environment via community structure [2]. Changes in the diversity of habitats wrought by allogenic forces and pollutants can be assessed using biotic or diversity indices [3]. Nature promotes diversity, whereas eutrophication increases dominance by one or a few species [4]. Since the application of single numerical indexesfor the determination of the community structure and ecological status of its ambient environment oversimplifies the real importance of its biodiversity, the literature suggests the use of multiple indices for diversity evaluation [5, 6, 7, 8, 9]. The theory of community diversity is based on two important features: the number of species, and the evenness of species [3, 6, 10]. To formulate an index that links these two features of diversity is a key challenge [11, 12, 13]. One of the key featuresof species diversity evaluation is that the basic constituents of most of the indices are associated with each other, and frequently participate together. In essence, diversity indices attempt to characterise the dataset on the abundance and number of species present in a communityintoa single number, i.e., the diversity index, from which community structure is hypothetically elucidated [9]. In an overall assessment, species diversity is a function of the number of species and theirrelative abundance. An increase in diversity requires a quintessential rise in the equitable distribution of species, even if the number of species decreases. Diversity is a significant feature of the community structure in which the presence of rare specieswould otherwise have been oflittle significance [14].

There are many diversity indices – Shannon, Simpson, Renyi, Weiner, etc. – which are used for determining the diversity and equitability of diverse communities [15, 16, 17, 18].

These indices have been widely applied by various workers for evaluating the communities in diverse ecosystem types [19, 20, 21, 22]. Biodiversity studies, in general, can be undertaken at three hierarchical levels:

i) Within-community species diversity (α-diversity);
ii) Change of diversity between communities (β-diversity); and
iii) Multi-community diversity (γ-diversity).

Alpha (α) diversity indicates the richness of species [23] at the level of individual communities, while beta (β) diversity represents the rate of species turnover [24, 25] between two adjoining communities. Gamma (γ) diversity represents the number of species in several adjoining communities, and at the landscape level [23]. Among all the indices at the level of α-diversity, Shannon's [15] index presents remarkable characteristics and is used extensively [26, 27, 28]. Simpson [16] developed the first index which indicates the probability of two randomly chosen individuals associating with the same species. Kempton and Taylor [29] proposed a new α-diversity index, the Q-statistic,which depends upon the quartiles of the species richness distributions. The first β-diversity index was developed by Whittaker in the 1960s [24]. Wilson and Mohler [30] developed another index that is dependent on the gradient length and the species turnover. Okland [31] used the ordination of sample plots to assess β-diversity with respect to the standard deviation of the species turnover.

α-diversity can be studied essentially for its two most important characteristics: dominance and diversity. Dominance is a measure of the information energy of a system, whereas diversity is the information entropy of a system. If a system consists of information which is concentrated in one or a few species, i.e., if one or a few species have the maximum number of individuals, then that system has more dominance. On the other hand, if a system shares information more or less equally among its species, i.e., the number of individuals of different species are equal or nearly equal, it has more diversity.

Most of the dominance and diversity indices that are being extensively used considerthe probability of occurrence of a species in a community, or a sample. A comprehensive look at the community organisation reveals the patterns of diversity on the following accounts:

i) Number of species –single-species communities vs. multispecies communities;
ii) Number of individuals of different species – dominant species vs. evenly distributed species;
iii) Average number of individuals per species; and
iv) Total number of individuals of all the species in the community – sparse community or a thick community.

Simpson's index of dominance and the sample variance share a common feature:both of these characteristics involve sums of squares in their formulae. In the case of Simpson's index, it is the sum of squares of probabilities, whereasin case of variance it is the mean sum of squares of deviations. Since the sum of squares of deviations can be converted to the sum of squares of probabilities, we should be able to derive Simpson's index fromthe sample variance. Furthermore, from the data on the variance of the number of individuals of different species within a community, we canalso develop new dominance and diversity indices. A review of the literature reveals that variance, standard deviation, standard error and coefficient of variation are the most frequently used statistics in biology and can be used to assess the dominance and diversity of communities. Diversity studies may also extend beyond the domain of biology into other research areas. For example, we can compare two languages on the basis of their information content using diversity indices. Development of diversity indices from descriptive statistics will give us a new tool to compare different systems based on their variabilities. This paper, therefore, attempts to derive new dominance and diversity indices based on the numerical strength of different species in a quadrat, or any other biological sample.

The new indices developed were computed for the simulated data and were also regressed on the dominance and diversity indices already in use. The problem was addressed as per the plan given below:

1. Derivation of the relationship between thevariance of a quadrat consisting of two or more species (*K* > 1) and Simpson's dominance index.
2. Sample variance,$S^{2}=\frac{\sum_{i=1}^{K}{\left(x_{i}-M\right)}^{2}}{K-1}\text{,}$ cannot be defined if (*K* = 1). The present paper envisages to define the variance of a sample for (*K* = 1).
3. Derivation of formulae for dominance and diversity indices from community/quadrat statisticsconsisting of one or more species (K ≥ 1).
4. To draw binary information plots of new derived indices.
5. To compute Shannon's, Simpson's and variance-based indices for simulated data on 25 quadrats.
6. To correlate newdominance and diversity indices developed with Shannon's and Simpson's indices.

## 2 Methods

The terms used in this paper are explained as follows:

### 2.1 Community

A community comprises of all the species (*K* species) present in an area, each species being represented by (*x*_*i*_) number of individuals. In statistical terms, a community can be treated as a population of size equal to *K*, and the number of individuals of a species can be considered as the number of observations (*x*_*i*_), or the values.

### 2.2 Quadrat

Community characterisation is generally carried out using sampling units called quadrats. A quadrat is a sample drawn from a community, and ideally represents the same species composition as that of the community consisting of *K* species.

It is presumed that each species in a quadrat hasthe number of individuals (*x*_*i*_) in the same proportion as in the community. In a statistical context, a quadrat is a sample of size *K*. The number of individuals of a speciesmay be considered as an observation (*x*_*i*_) of a sample. In this paper, in order to avoid the multiplicity of terms, *K* and *x* have been used as the number of species and number of individuals per species, respectively, both for a community and a quadrat. In particular, for a single-species community or quadrat, *K* is equal to 1. The indices derived from quadrats are generally known as community indices.

### 2.3 Mean number of individuals

The mean number of individuals per species in a community, in statistical terms, is a population parameter, whereas for a quadrat, it is a statistic. In this paper both the meanshave been represented as *M*.

### 2.4 Measures of dispersion

In statistical terms, the variance of the number of individuals of different species (*x*_*i*_) around their mean *M*in a communityis a parameter and is represented as (*σ*^2^). For a quadrat (sample) it will be a statistic (*Var* or *S*^2^). Similarly, notations used for the standard deviation of a community anda quadrat are (*σ*) and (*S*) respectively. *SE* and *CV* represent standard error and coefficient of variation of the number of individuals of different species in a quadrat, respectively. Notations used in statistics and community analysis are given in Table 1.

**Table 1:** Terminology used in statistics and community analysis.

Population	Sample	Community	Quadrat
Population size (*K*)	Sample size (*K*)	Number of spp. (*K*)	Number of spp. (*K*)
Observations or Values (*x*_*i*_)	Observations or Values (*x*_*i*_)	Number of individuals of a sp. (*x*_*i*_)	Number of individuals of a sp. (*x*_*i*_)
Population mean (*M*)	Sample Mean (*M*)	Mean number of individuals per sp. (*M*)	Mean number of individuals per sp. (*M*)
Population variance (σ^2^)	Sample variance (*S*^2^)	Community variance (σ^2^)	Quadrat variance (*S*^2^ or *Var*)
Population standard deviation (σ)	Sample standard deviation (*S*)	Community standard deviation (σ)	Quadrat standard deviation (*SD*)

### 2.5 Maximum variance

The variance of a sample varies with its composition. A theoretical statistic, *Var*(*max*), has been defined as the maximum variancethat a sample can have. Theoretically, a sample of size *K* and mean *M* will have the maximum variance if one of its elements is assigned the sum of all the values, $\overset{K}{\underset{i=1}{\sum }}x_{i}$, and all other elements are zeros. Such a sample has been named as a single-valuevariable. Any sample, with the same size (*K*) and mean (*M*), will have a variance between 0 (when all values are equal) and *Var*(*max*) (single-valuevariable). An example is given in Table 2.

**Table 2:** Example of a computation of *Var*(*max*) using theoretic single value variable.

Sample	Quadrat	Sample I	Sample II	Sample III
		All values equal	Single value variable	Experimental Data
Sample size (*K*)	Number of species	5	5	5
Values (*x*_*i*_)	Number of individuals of different species	2, 2, 2, 2, 2	10, 0, 0, 0, 0	2, 4, 1, 2, 1
Mean (*M*)	Mean number of individuals per species	2	2	2
Sample variance (*S*^2^) or *Var*	Quadrat variance	0 *Var*(*min*)	20 *Var*(*max*)	1.5

### 2.6 Shannon-Weiner's and Simpson's indices

The probability of occurrence of individuals of a species in a community or a quadrat ($p_{i}$) is given as,

$$p_{i}=\frac{Number\;of\;individuals\;of\;a\;species\;in\;a\;community\;or\;a\;quadrat}{Total\;number\;of\;individuals\;of\;all\;the\;species\;in\;the\;community\;or\;quadrat}$$

Shannon's and Simpson's indices were computed using the equations as given below:

Shannon's diversity index, $H'=-\overset{K}{\underset{i=1}{\sum }}p_{i}\;ln\;p_{i}$

Simpson's dominance index,$C=\overset{K}{\underset{i=1}{\sum }}p_{i}^{2}$

Simpson's diversity index,$C'=1-\overset{K}{\underset{i=1}{\sum }}p_{i}^{2}$

### 2.7 Data for simulation studies

In order to understand the effectiveness and utility of the newly developed indices, data were simulated for 25 quadrats, with each quadrat consisting of 10 species and 100 individuals (Table 3) in different proportions so as to mimica wide range of community characteristics – from a community dominated byone species, to a communityconsisting of tenevenly distributed species. Dominance and diversity indices were generated for all of the quadrats, andlinear, non-linear and Spearman's rank correlations were calculated to correlate the newly developed indiceswith the commonly used indices, using PAST-3 and MS-Excelsoftware.

**Table 3:** Data used for 25 simulated samples with 10 species (Sp.) and 100 individuals per sample to find correlations among with various dominance and diversity indices.

Number of individuals in quadrats										
Quad.	Sp.1	Sp.2	Sp.3	Sp.4	Sp.5	Sp.6	Sp.7	Sp.8	Sp.9	Sp.10
Q1	10	10	10	10	10	10	10	10	10	10
Q2	91	1	1	1	1	1	1	1	1	1
Q3	35	3	26	8	9	5	4	3	5	2
Q4	3	5	4	2	1	2	2	70	8	3
Q5	1	13	4	9	20	22	12	4	5	10
Q6	32	1	2	12	23	2	23	2	2	1
Q7	1	2	4	23	8	2	3	46	10	1
Q8	1	32	7	23	1	1	12	2	12	9
Q9	31	22	17	4	7	5	4	3	5	2
Q10	8	3	13	24	12	12	5	12	9	2
Q11	8	9	12	18	21	4	12	6	2	8
Q12	3	6	5	3	23	11	15	17	5	12
Q13	12	24	3	29	3	6	8	5	7	3
Q14	2	3	7	6	1	51	5	11	12	2
Q15	4	34	2	2	3	7	37	6	3	2
Q16	1	3	13	12	25	21	2	4	12	7
Q17	7	12	32	4	7	4	5	8	7	14
Q18	1	4	66	3	4	1	13	4	2	2
Q19	12	19	9	3	3	12	5	1	4	32
Q20	2	22	44	1	2	6	5	3	4	11
Q21	4	6	27	18	20	1	6	2	11	5
Q22	2	3	17	4	7	3	4	16	1	43
Q23	3	3	9	8	21	11	1	24	14	6
Q24	3	4	45	6	23	2	1	1	8	7
Q25	54	13	2	1	6	4	2	1	9	8

## 3 Results

### 3.1 Population variance as ameasure of information

Consider a large community consisting of *K* species, with *x*_*i*_ representing the number of individuals of different species. Let *M* be the mean number of individuals per species in the community; then the variance of the community (σ^2^) may be treated as population variance and may be defined as per Eq. (1) [32]

(1) $$\sigma^{2}=\frac{\sum_{i=1}^{K}{\left(x_{i}-M\right)}^{2}}{K}\ldots \left(K\geq 1\right)\text{.}$$

Parkash and Thukral [26] proved that variance is a measure of information as given by Simpson's concentration. In Eq. (1), dividing and multiplying (*x*_*i*_) with the total number of individuals of all species in the community ($\overset{K}{\underset{i=1}{\sum }}x_{i}$) we get,

$$\sigma^{2}=\frac{\sum_{i=1}^{K}{\left(\left(\frac{x_{i}}{\sum_{i=1}^{K}x_{i}}\sum_{i=1}^{K}x_{i}\right)-M\right)}^{2}}{K}\ldots \left(K\geq 1\right)\text{.}$$

Substituting$\frac{x_{i}}{\sum_{i=1}^{K}x_{i}}=p_{i}\text{,}$and$\overset{K}{\underset{i=1}{\sum }}x_{i}=KM\text{,}$we get

$$\sigma^{2}=\frac{\sum_{i=1}^{K}{\left(p_{i}KM-M\right)}^{2}}{K}.$$

Simplifying the equation, we get,

(2) $$\sigma^{2}=M^{2}\left(K\overset{K}{\underset{i=1}{\sum }}p_{i}^{2}-1\right)\ldots \left(K\geq 1\right).$$

Eq. (2) proves that population variance ($\sigma^{2}$) is a measure of information ($\overset{K}{\underset{i=1}{\sum }}p_{i}^{2}$).

### 3.2 Derivation of Simpson's indices from population variance

In terms of community characterisation, Simpson's index of dominance,$\;C=\overset{K}{\underset{i=1}{\sum }}p_{i}^{2}$, (Simpson, 1948) is based on the probability that two individuals drawn from a community belong to the same species. From Eq. (2) we find that the variance-to-mean square ratioof individuals of different species is a linear function of Simpson's index of dominance.

$$\frac{\sigma^{2}}{M^{2}}=K\overset{K}{\underset{i=1}{\sum }}p_{i}^{2}-1\ldots \left(K\geq 1\right).$$

Vice-versa, Simpson's indices of dominance (*C*)and diversity (*C*′) can be derived from the community parameters of variance, number of species and the number of individuals per species.

$$C=\overset{K}{\underset{i=1}{\sum }}p_{i}^{2}=\frac{\sigma^{2}}{KM^{2}}+\frac{1}{K}\ldots \left(K\geq 1\right).$$

Putting the values of *K* = 1, and *x*_1_ = *M*in Eq. (1), σ^2^ is equal to 0. Therefore, as per the equation given above, Simpson's index of dominance will be equal to 1. Simpson's index of diversity (*C′*) [20, 33] is given below:

$$C^{'}=1-\overset{K}{\underset{i=1}{\sum }}p_{i}^{2}=1-\left(\frac{\sigma^{2}}{KM^{2}}+\frac{1}{K}\right)\ldots \left(K\geq 1\right).$$

If the variance of a large community consisting of only one species (*K* = 1) is zero, then Simpson's index of diversity will also be zero (*C*’ = 0).

### 3.3 Derivation of Simpson's index from sample variance for K > 1

Phytosociological studies are generally conducted using samples called quadrats. Let a quadrat be taken froma community which represents the same proportions of individuals of species as in the community.

Then, the variance (*Var*, or *S*^2^) of number of individuals (*x*) of different species in the quadrat, as per the definition of sample variance will be

(3) $$S^{2}=\frac{\sum_{i=1}^{K}{\left(x_{i}-M\right)}^{2}}{K-1}\ldots \left(K>1\right),$$

where *M* represents the average number of individuals per species, and *K* is the number of species present in the quadrat. We know that the sample variance (*S*^2^) is an unbiased estimator of population variance (σ^2^) [34,35]:

$$\sigma^{2}=\frac{\left(K-1\right)}{K}S^{2}.$$

From Eqs. (2) and (3) we get,

(4) $$Var=S^{2}=\frac{KM^{2}}{K-1}\left(K\overset{K}{\underset{i=1}{\sum }}p_{i}^{2}-1\right)\ldots \left(K>1\right).$$

As shown in Eq. (4)$\overset{K}{\underset{i=1}{\sum }}p_{i}^{2}<1$, the variance of a quadrat consisting of more than one species will be less than that of a quadrat consisting of a single species. Rearranging Eq. (4), Simpson's index of dominance may be obtained for quadrat statistics

$$\overset{K}{\underset{i=1}{\sum }}p_{i}^{2}=\left(\frac{K-1}{K^{2}}\right)\frac{S^{2}}{M^{2}}+\frac{1}{K}\ldots \left(K>1\right).$$

### 3.4 Derivation of sample variance for*K* = 1

As per Eq. (3), the variance of a sample cannot be defined for *K* = 1. In order to derive dominance and diversity indices from sample variance, it is necessary to define the variance of a sample with a single observation. Since the mean of a single value is the value itself, it is evident that as the value of *K* approaches 1, the value of *x*_*i*_ approaches the mean, and the equation assumes an indeterminate form, i.e., the variance (*S*^2^) approaches 0/0.

Then, as per Eq. (4), the maximum variance of a sample will be obtained if the value of$\overset{K}{\underset{i=1}{\sum }}p_{i}^{2}=1$. Assume that a sample consists of*K*observations:$\left[x_{1},x_{2},\;x_{3\;},\ldots ,x_{K}\right]$, such that $\overset{K}{\underset{i=1}{\sum }}x_{i}\;\neq 0$. Then, numerically we can prove that among all the samples of size *K*,a sample consisting of values in the form,$\left[\overset{K}{\underset{i=1}{\sum }}x_{i},0,\;0,\ldots ,0\right]$, will have the maximum variance. Putting the value of$\overset{K}{\underset{i=1}{\sum }}p_{i}^{2}=1$ in Eq. (4) gives us an equation for the maximum variance,

(5) $$Var\left(\max\right)=\frac{KM^{2}}{\left(K-1\right)}\left(K-1\right)=KM^{2}\ldots \left(K\geq 1\right).$$

Eq. (5) is a result of (*K*-1)in the denominator, cancelling out (*K*-1) in the numerator. This leads us to an important result to define the variance of a sample for *K* = 1,

(6) $$Var\left(\max)=Var=M^{2}=x_{1}^{2}\ldots (K=1\right).$$

Thus, the variance of a sample consisting of a single value is equal to the square of the value. The result in Eq. (6) can also be proved fromthegraphical analysis of data given in Table 4. In order to define the variance of a single observation, let us define a single-valuevariable (*X*) in which the first value of *x* > 0, and all other values are zeros. That is,

$$X:\left[x_{1},0,0,\ldots ,0\right]\ldots \left(x_{1}>0\right).$$

**Table 4:** Sample statistics for single value variables of different sample sizes (number of species, *K* > 1), with *x*_1_ > 0, and other sample values equal to 0, to extrapolate the variance/mean square ratio for *K* = 1.

Sample size, (number of species, *K*)	2	3	4	5	6	7	8
*x*_1_	10	10	10	10	10	10	10
*x*_2_	0	0	0	0	0	0	0
*x*_3_		0	0	0	0	0	0
*x*_4_			0	0	0	0	0
*x*_5_				0	0	0	0
*x*_6_					0	0	0
*x*_7_						0	0
*x*_8_							0
Mean(M)	5	3.33	2.5	2	1.66	1.42	1.25
Var(Max)	50	33.33	25	20	16.66	14.28	12.5
$\frac{Var\left(Max\right)}{M^{2}}$	2	3	4	5	6	7	8
SD	7.07	5.77	5	4.47	4.08	3.77	3.53
SE	5	3.33	2.5	2	1.66	1.42	1.25

*Var*(*Max*) is achieved when one of the values of *x*_1_ > 0, and other *x* values are zeroes. SD = Standard deviation, SE = Standard error. The value 10 is tentative. Any value of *x*_1_ > 0 will give the *Var*(*Max*)/*M*^2^ ratio equal to *K*.

We can find the variance of a sample for *K* = 1 by extrapolation. A variable will have the maximum variance, *Var*(*max*), if, $\overset{K}{\underset{i=1}{\sum }}p_{i}^{2}=1.$Among all the possible variables with the same *K* and *M*, the single-valuevariable will have the maximumsample variance, *Var*(*max*). For example, let us define different variables with *K* = 4, and *M* = 5,

X:[20,0,0,0];A:[10,5,5,0];B:[8,5,4,3];C:[5,4,2,9];etc.

If we compute the variances of the samples given above andother similar variables, the single-valuevariable (*X*), the first one given above, will have the maximum sample variance.

We can find the sample variances of single-value variables for different values viz., *K* = 2, 3, 4, etc. Table 4 gives the single-value variables, and their variance/mean ratios. It is seen in the Table that, for a single-valuevariable, the following relationship holds,

$$\frac{Var\left(\max\right)}{M^{2}}=K\ldots \left(K>1\right).$$

**Fig. 1:**
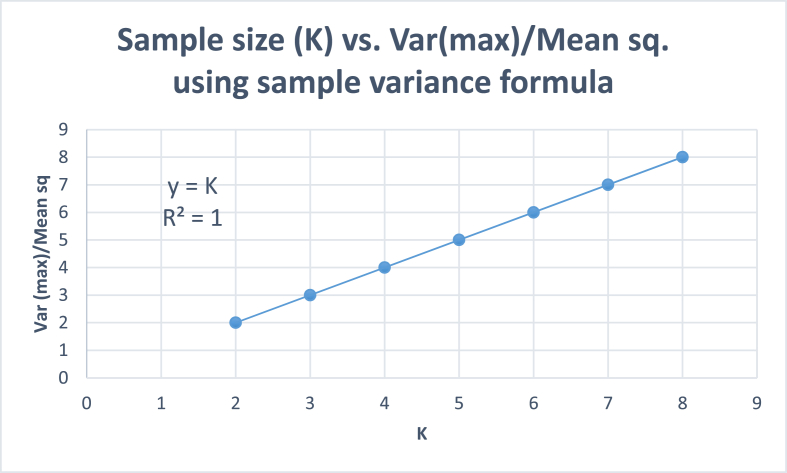
Theoretical plot between sample size (*K*) and Var/Mean square ratio for quadrats in which all the individuals belong to one species using sample variance formula.

Fig. 1 gives *Var*(*max*)/*M*^2^ ratios for *K* > 1. We can jointhese points to get a straight line with a slope equal to 1. Extrapolating this straight lineto *K* = 1 gives the value of *Var*(*max*)/*M*^2^as equal to 1. This gives us a graphical method to provethat the variance of a variableconsisting of only a singlevalue, *x* > 0, is equal to *x*^2^. Using this method, we can find the variances of single-species communities and quadrats, which we proved mathematically in Eq. (6).

### 3.5 Population variance for *K* = 1

On the other hand, using the population variance formula (Eq. 2), we get,

$$\frac{\sigma_{\max}^{2}}{M^{2}}=K\sum_{i=1}^{K}p_{i}^{2}-1\ldots \left(K\geq 1\right).$$

For$\sum_{i=1}^{K}p_{i}^{2}=1,$ $\frac{\sigma_{\max}^{2}}{M^{2}}=K-1$,and

$$\sigma_{\max}^{2}=0\ldots \left(K=1\right).$$

### 3.6 Properties of sample variance with *K* = 1

Sample variance for *K* = 1 follows the important properties of sample variance for*K*>1.

#### 3.6.1 Multiplication of the variable (*K* = 1) with a constant

Let *X* and *Y* be random variables, and *c* be a constant. Then, the variance of (*cX*) will be [34],

$$Var\left(cX\right)=c^{2}Var\left(X\right)\;...\;\left(K>1\right).$$

For a sample with *K* = 1,

$$Var\left(cX\right)=c^{2}M^{2}=c^{2}Var\left(X\right)\ldots \left(K=1\right).$$

#### 3.6.2 Covariance between two variables for *K* = 1

Covariance is given by,

$$Covar\left(X,Y\right)=\frac{\sum_{i=1}^{K}\left(x_{i}-M_{x}\right)\left(y_{i}-M_{y}\right)}{K-1},$$

where *M*_*x*_ and *M*_*y*_ are the means of *X* and *Y,* respectively. On simplifying we get,

$$Covar\left(X,Y\right)=\frac{\sum_{i=1}^{K}\left(x_{i}y_{i}\right)-K\frac{\sum_{i=1}^{K}x_{i}\sum_{i=1}^{K}y_{i}}{K^{2}}}{K-1}.$$

For single-value-variables *X* and *Y*, we get

$$Covar\left(X,Y\right)=\frac{\sum_{i=1}^{K}\left(x_{i}y_{i}\right)-\frac{\sum_{i=1}^{K}x_{i}\sum_{i=1}^{K}y_{i}}{K}}{K-1}$$

$$=\frac{K^{2}M_{x}M_{y}-\frac{K^{2}M_{x}M_{y}}{K}}{K-1}$$

$$=\frac{K^{2}M_{x}M_{y}-KM_{x}M_{y}}{K-1}$$

$$=\frac{KM_{x}M_{y}\left(K-1\right)}{K-1}=KM_{x}M_{y}.$$

(7) $$\text{Thus},\text{ ​for ​K}=1,\text{ ​}Covar\left(X,Y\right)=M_{x}M_{y}\text{.}$$

#### 3.6.3 Variance of sum of two variables (*K* = 1)

The variance of sum of variables *X* and *Y* for *K* > 1 is [34, 36]

Var(X+Y)=Var(X)+Var(Y)+2Covar(X,Y),

where *Covar* is the sample covariance between *X* and *Y*.

$$Var\left(X+Y\right)=M_{x}^{2}+M_{y}^{2}+2M_{x}M_{y}={\left(M_{x}+M_{y}\right)}^{2}\;...\;\left(K=1\right)$$

**Fig. 2:**
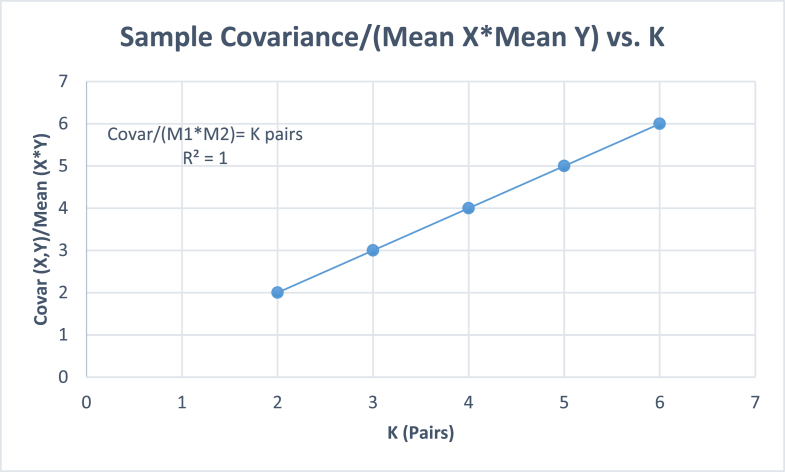
Graph to extrapolate the sample covariance of single value variables.

Table 5 and Fig. 2 give the covariance of single-valuevariables *X* and *Y*, with their first values being more than 0, the other values being zeros. On extrapolation to *K* = 1, the straight line gives covariance equal to the product of their values.

**Table 5:** Sample covariance between pairs of single value variables, *X* and *Y*, with one of the values of *x*_*i*_ > 0, and the other values of *x* equal to zeroes.

Sample size, (number of species, *K*)	*K* = 2		*K* = 3		*K* = 4		*K* = 5		*K* = 6	
Samples (*X,Y*)	*X*	*Y*	*X*	*Y*	*X*	*Y*	*X*	*Y*	*X*	*Y*
Species	Number of individuals									
*x*_1_ > 0	10	4	10	4	10	4	10	4	10	4
*x*_2_	0	0	0	0	0	0	0	0	0	0
*x*_3_			0	0	0	0	0	0	0	0
*x*_4_					0	0	0	0	0	0
*x*_5_							0	0	0	0
*x*_6_									0	0
Mean (*M*)	5	2	3.33	1.33	2.5	1	2	0.8	1.66	0.66
Covar.(X,Y)	20		13.33		10		8		6.66	
$Covar/\left(M_{X},M_{Y}\right)$	2		3		4		5		6	
Regr.(X,Y)	0.4		0.4		0.4		0.4		0.4	

The values 10 and 4 are tentative. Any set of values of *x*_1_ > 0 will give the *Covar*/(*M*_1_*M*_2_) ratio equal to *K*.

#### 3.6.4 Correlation and regression between two variables (*K*1)

Linear correlation between two variables, X and Y is given as,

$$Correl.\;\left(X,Y\right)=\frac{Covar\;\left(X,Y\right)}{\sqrt{Var\left(X\right)\;Var\left(Y\right)}}\;,$$

where *Var*(*X*) and *Var*(*Y*)are variances of *X* and *Y*. From Eqs. (6) and (7),

$$Correl.\;\left(X,Y\right)=\frac{M_{x}M_{y}}{\sqrt{M_{x}^{2}M_{y}^{2}}}=1\ldots \left(K=1\right).$$

Correlation between two single-valuevariables for *x*_1_ > 0 and *y*_1_ >, 0 is equal to 1, both empirically and by derivation. If one of the mean values is negative, the correlation is -1. The same holds true for *K=*1.

Linear regression of *Y* on *X* is,

$$Regression\;\left(X,Y\right)=\frac{Covar\;\left(X,Y\right)}{Var\left(X\right)}$$

From Eqs. (6) and (7), we get,

$$Reg.\;\left(X,Y\right)=\frac{M_{x}M_{y}}{M_{x}^{2}}=\frac{M_{y}}{M_{x}}\ldots \left(K=1\right).$$

Regression between two single-valuevariables for *x*_1_ > 0 and *y*_1_ > 0, is equal to theratio of means of *Y* and *X*, both empirically and by derivation. The same holds for *K=*1.

### 3.7 Derivation of variance-based dominance and diversity indices

We have proved that the variance of a sample for *K* = 1 is equal to the square of its value. Therefore, we can calculate the variance of a single-species community for the purpose of development of dominance and diversity indices based on sample statistics. Simpson's index of dominance may be given as,

$$\overset{K}{\underset{i=1}{\sum }}p_{i}^{2}=\left(\frac{K-1}{K^{2}}\right)\frac{Var}{M^{2}}+\frac{1}{K}\ldots \left(K\geq 1\right).$$

Gini-Simpson's index of diversity can be obtained as follows:

$$1-\overset{K}{\underset{i=1}{\sum }}p_{i}^{2}=1-\left[\left(\frac{K-1}{K^{2}}\right)\frac{Var}{M^{2}}+\frac{1}{K}\right]\ldots \left(K\geq 1\right)$$

$$=\frac{K-1}{K}\left(1-\frac{Var}{KM^{2}}\right)\ldots \left(K\geq 1\right).$$

Therefore, from the equations derived above, we can propose new dominance and diversity indices for a quadrat or any other ecological sample. Variance of a sample can be written as,

(8) $$Var=\frac{KM^{2}}{K-1}\left(K\overset{K}{\underset{i=1}{\sum }}p_{i}^{2}-1\right)\ldots \left(K>1\right)$$

$$Var=M^{2}\ldots \left(K=1\right)$$

$$Var\left(\max\right)=KM^{2}\ldots \left(K\geq 1\right).$$

### 3.8 Relationship between variance-based dominance and diversity indices

**Fig. 3:**
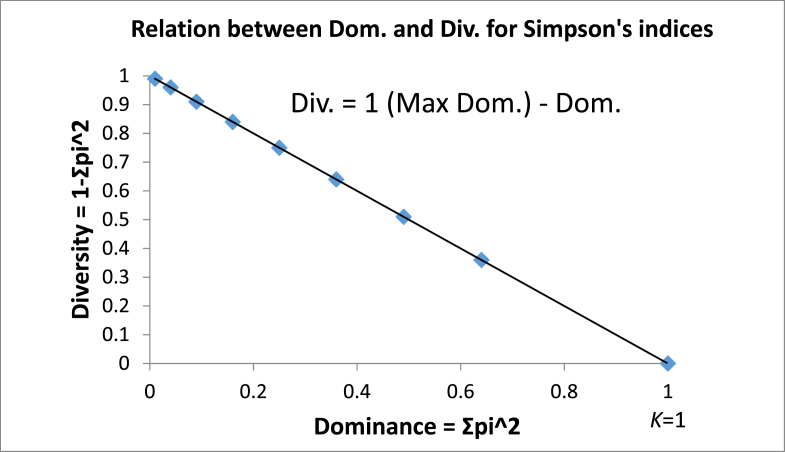
Graph between Simpson's Dominance and Diversity indices.

The dominance and diversity indices for a quadrat consisting of *K* species and *M* number of individuals per species can be defined as a function of variance. Diversity is equal to the difference between the maximum dominance for a single-value variable minus the actual dominance. Asdominance increases, diversity decreases (Fig. 3). Dominance will be at maximumfor *K* = 1, whereasthe diversity of a community or a quadratconsisting of more than one species will be at maximum if all the species have an equal number of individuals. The dominance-diversity equation is,

*Diversity* = *Dominance* (*max*) – *Dominance* (*actual*)

From Eq. (8), the variance-to-mean square ratio is a linear function of Simpson's index and can be used as an index of dominance.

$$\frac{Var}{M^{2}}dominance\;index=\frac{Var}{M^{2}}\ldots \left(K\geq 1\right).$$

Since,

$$\frac{Var}{M^{2}}\left(\max\right)=K\ldots \left(K\geq 1\right),$$

we can derive the variance-to-mean square ratio index of diversity,

$$\frac{Var}{M^{2}}diversity\;index=K-\frac{Var}{M^{2}}\ldots \left(K\geq 1\right).$$

For a single-species quadrat (*K=*1) and*Var* = *M*^2^, the *Var/M*^2^ dominance index will be 1, and the complementary*K*-*Var/M*^2^ diversity index will be equal to 0. The new indices of dominance and diversity, as derived from variance and standard deviation, are given in Tables 6 and 7.

**Table 6:** Dominance indices developed from quadrat statistics.

Name of the Dominance index	Statistic used as Dominance index	Information equation for Dominance index	Max. Dom. for K spp.	Dom. for single sp.	Dom. for evenly distributed spp.	Comments
Variance Dominance index	*Var*	$\frac{KM^{2}}{K-1}\left(K\overset{K}{\underset{i=1}{\sum }}p_{i}^{2}-1\right)$	*KM*^2^	*M*^2^	0	High scale of dominance. Not preferred.
Variance to mean square ratio Dominance index	$\frac{Var}{M^{2}}$	$\frac{K}{K-1}\left(K\overset{K}{\underset{i=1}{\sum }}p_{i}^{2}-1\right)$	*K*	1	0	Gives dominance on a scale of number of spp.
Variance to mean ratio Dominance index	$\frac{Var}{M}$	$\frac{KM}{K-1}\left(K\overset{K}{\underset{i=1}{\sum }}p_{i}^{2}-1\right)$	*KM*	*M*	0	Dominance on a scale of number of individuals. Useful for sparse communities.
Variance to mean square per species Dominance index	$\frac{Var}{KM^{2}}$	$\frac{1}{K-1}\left(K\overset{K}{\underset{i=1}{\sum }}p_{i}^{2}-1\right)$	1	1	0	Dominance on a scale of 0–1. Most useful. Similar to Simpson's dominance.
Variance to mean per species Dominance index	$\frac{Var}{KM}$	$\frac{M}{K-1}\left(K\overset{K}{\underset{i=1}{\sum }}p_{i}^{2}-1\right)$	*M*	*M*	0	Dominance scale of 0 to mean number of individuals per species.
Variance per species Dominance index	$\frac{Var}{K}$	$\frac{M^{2}}{K-1}\left(K\overset{K}{\underset{i=1}{\sum }}p_{i}^{2}-1\right)$	*M*^2^	*M*^2^	0	Large scale of 0 to number of individuals per species squared.
Standard deviation Dominance index	*SD*	$M\sqrt{\frac{K}{K-1}\left(K\overset{K}{\underset{i=1}{\sum }}p_{i}^{2}-1\right)}$	*M√K*	*M*	0	High scale variability. Useful for sparse communities.
Coefficient of variation Dominance index	*CV*	$\sqrt{\frac{K}{K-1}\left(K\overset{K}{\underset{i=1}{\sum }}p_{i}^{2}-1\right)}$	√*K*	1	0	Scale of 0 to square root of species.
Standard error Dominance index	*SE*	$\frac{M}{\sqrt{K}}\sqrt{\frac{K}{K-1}\left(K\overset{K}{\underset{i=1}{\sum }}p_{i}^{2}-1\right)}$	*M*	*M*	0	Scale of 0 to mean number of individuals per species.
Simpson's Dominance index	*p*	$C=\overset{K}{\underset{i=1}{\sum }}p_{i}^{2}$	1	1	$\frac{1}{K}$	Varies from 1 to 1/K. Most commonly used index.

K= Number of species, M = Mean number of individuals per species, Var = variance, SD = Standard deviation, CV = Coefficient of variation, SE = Standard error, *p* = probability of occurrence of a species.

**Table 7:** Diversity indices developed from quadrat statistics.

Name of the Diversity index	Statistic used as Diversity index	Div. for single sp. community	Div. for evenly distributed spp. community	Comments
Variance Diversity index	$KM^{2}-Var$	0	$KM^{2}$	Gives a very high value for evenness. May not be preferred.
Variance to mean square ratio Diversity index	$K-\frac{Var}{M^{2}}$	0	*K*	Gives diversity on a scale of 0 to number of species. To some extent comparable to Shannon's evenness index, Exp (*H′*).
Variance to mean ratio Diversity index	$KM-\frac{Var}{M}$	0	*KM*	Ranges from 0 to number of individuals in a sample. Useful for sparse communities.
Variance to mean square per species Diversity index	$1-\frac{Var}{KM^{2}}$	0	1	Defines diversity on a scale of 0–1. Best to use.
Variance to mean per species Diversity index	$M-\frac{Var}{KM}$	0	*M*	Diversity on a scale of 0 to mean number of individuals per species.
Variance per species Diversity index	$M^{2}-\frac{Var}{K}$	0	*M*^2^	0 to squared mean number of individuals per species.
Standard deviation Diversity index	$M\sqrt{K}-SD$	0	$M\sqrt{K}$	Defines diversity on the basis of standard deviation units.
Coefficient of variation Diversity index	$\sqrt{K}-CV$	0	*√K*	Complementary to square root number of species. Good to use.
Standard error Diversity index	*M – SE*	0	*M*	Scale of 0 to mean number of individuals per species.
Simpson's Diversity index	$C^{'}=1-\overset{K}{\underset{i=1}{\sum }}p_{i}^{2}$	0	$1-\frac{1}{K}$	Complementary to Simpson's dominance. Commonly used.
Simpson's Reciprocal diversity index	$\frac{1}{C}=\frac{1}{\sum_{i=1}^{K}p_{i}^{2}}$	1	*K*	Reciprocal of Simpson's dominance. Widely used.
Shannon's Diversity index	$H^{'}=-\overset{K}{\underset{i=1}{\sum }}p_{i}\;\ln p_{i}$	0	Ln *K*	Shannon's entropy. Widely used.
Shannon's evenness index	Exp (*H′*)	1	*K*	Gives the evenness of distribution of species.

K= Number of species, M = Mean number of individuals per species, Var = variance, SD = Standard deviation, CV = Coefficient of variation, SE = Standard error, p = probability of occurrence of a species.

### 3.9 Dominance and diversity for even distribution of species

To determine the dominance and diversity of quadrats consisting of equal numbers of individuals of allspecies, (*x*_1_ = *x*_2_ … = *x*_K_),Simpson's index of dominance is,

$$\overset{K}{\underset{i=1}{\sum }}p_{i}^{2}=\overset{K}{\underset{1}{\sum }}\frac{x_{i}^{2}}{{\left(\sum_{1}^{K}x_{i}\right)}^{2}}=\frac{1}{K}...\left(x_{1}=x_{2}=\ldots =x_{K}\right).$$

Similarly, for a community with an equal number of individuals in allspecies, we have,

$$\frac{Var}{M^{2}}=\frac{K}{K-1}\left(K\overset{K}{\underset{i=1}{\sum }}p_{i}^{2}-1\right)=\frac{K}{K-1}\left(K\frac{1}{K}-1\right)=0\ldots \left(K\geq 1\right).$$

**Fig. 4:**
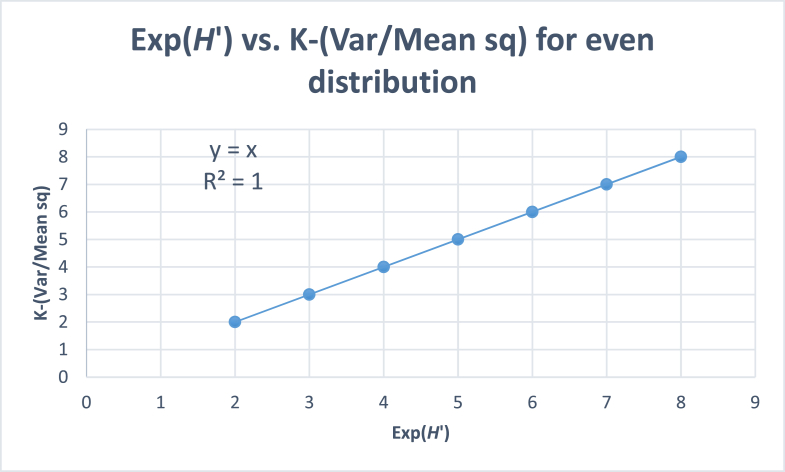
Plot between Exp (*H′*) and (K-Var/Mean sq) for all species in a quadrat having equal number of individuals.

Table 8 gives variance/mean square ratios for samples with equal numbers of individuals for each species. It is seen that irrespective of the number of individuals of a species in a sample, the variance/mean square ratio is always zero. On the otherhand,*K*-(*Var/M*^2^)is always equal to the number of species in the sample. Since sample variance for a singlespecies cannot be calculated using the conventional variance formula with a denominator equal to (*K*-1), an extrapolation of data reveals that *K*-(*Var/M*^2^) for a sample with a single observation is equal to 1. A graph between Exp (*H'*) and *K*-(*Var/M*^2^) gives a straight line with a slope equal to *K* (Fig. 4).

**Table 8:** Sample Variance/Mean square ratio for different simulated sample sizes (number of species, *K*) with equal distribution of species.

Species (*K*)	2	3	4	5	6	7	8
	Number of individuals (*x*_*i*_)						
*x*_1_	1	2	10	45	68	100	132
*x*_*2*_	1	2	10	45	68	100	132
*x*_3_		2	10	45	68	100	132
*x*_4_			10	45	68	100	132
*x*_5_				45	68	100	132
*x*_*6*_					68	100	132
*x*_*7*_						100	132
*x*_*8*_							132
Mean (*M*)	1	2	10	45	68	100	132
	Dominance indices for even distribution of species						
Simpson's *C*	½	1/3	¼	1/5	1/6	1/7	1/8
*Var*	0	0	0	0	0	0	0
$\frac{Var}{M^{2}}$	0	0	0	0	0	0	0
	Diversity indices for even distribution of species						
Simpson's 1/*C*	2	3	4	5	6	7	8
$K-\frac{Var}{M^{2}}$	2	3	4	5	6	7	8
*H′*	0.693	1.098	1.386	1.609	1.791	1.945	2.079
Exp (*H*′)	2	3	4	5	6	7	8

The number of individuals of a species are simulated to demonstrate the universality of the statistic. A sample with equal number of individuals for all the species will give K-(Var/M^2^) = K.

### 3.10 Binary informationplots for dominance and diversity

Information within a system can be studied at two levels: information energy, $=\overset{K}{\underset{i=1}{\sum }}p_{i}^{2}$, as described by Onisescu [37], and information entropy $H'=-\overset{K}{\underset{i=1}{\sum }}p_{i}\;ln\;p_{i}$ as described by Shannon [15]. Information, as contained in dominance, is a measure of energy, whereas diversity is a measure of entropy. If information is plotted against a two-class variable, with probabilities *p* and (1-*p*), it gives a binary information plot.

Simpson's index of dominance is a measure of the energy of a system and gives a convex curve against probability, whereas Shannon's index of diversity is a measure of entropy within a system and gives a concave curve [38].

**Fig. 5:**
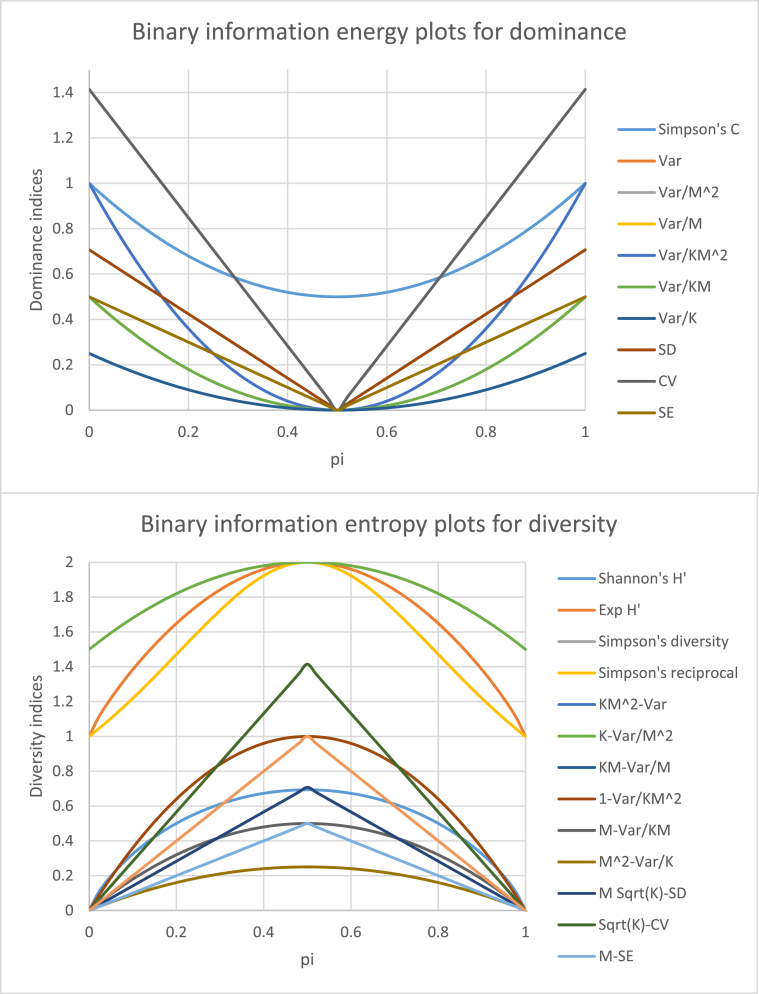
Binary plots between probability and dominance, and diversity indices. For dominance, curves for Simpson's C, Var, Var/M^2^, Var/M, Var/KM^2^, Var/KM, and Var/K are convex. The curves for Var, Var/M^2^, and Var/KM overlap. Similarly, curves for Var/M, Var/KM^2^ overlap with each other. For diversity, curves for Shannon's *H*′, Exp (*H*′), Simpson's diversity, Simpson's reciprocal, KM^2^-Var, K-Var/M^2^, KM-Var/M, 1-Var/KM^2^, M-Var/KM, and M^2^-Var/K are concave. Simpson's diversity, K M^2^-Var, and M-Var/KM overlap. Similarly, curves for KM-Var/M, 1-Var/KM^2^ overlap with each other.

If we consider a community with only two species, if the probability of one species is *p*, the probability of the other species will be (1-*p*). Fig. 5 gives dominance and diversity plots of the new indices developed *vis-à-vis* the commonly used Shannon's and Simpson's indices.

Simpson's measure of dominance is ameasure of information energy and gives a convex curve for its binary information plot (Fig. 5). Similarly, the curves for *Var*, *Var/M*^2^, *Var/M*, *Var/*(*KM*^2^), *Var*/(*KM*), and *Var/K* are also convex and are also information measures. The other three statistics, *SD*, *SE* and *CV*, however, give convex vertices and are not information measures. Nevertheless, as these indices have been derived from variance, these can also be used for dominance statistics. Fig. 5 also gives binary information plots for the diversity indices proposed vis-à-vis Simpson's index. We know that the curves for Shannon's index of diversity (*H′*), Exp (*H′*), Gini-Simpson's diversity and Simpson's reciprocal diversity indices are concave and areinformation measures. Similarly, the binary probability plots for (*KM*^2^-*Var*), (*K*-*Var*/*M*^2^), *KM*-(*Var/M*), 1-(*Var/KM*^2^), *M*-(*Var/KM*), and *M*^2^-(*Var/K*) are also concave curves. However, the graphs for diversity indices proposed using *SD*, *SE* and *CV* are concave vertices and are not information measures, although they can also be used as diversity indices.

### 3.11 Correlating new indices with Shannon's and Simpson'sindices

It was found that all the new dominance and diversity indices developed have significant positive correlations with Simpson's and Shannon's indices (Table 9). Variance-based dominance indices were positively and linearly correlated with Simpson's dominance index with correlation coefficients equal to one.

**Table 9:** Pearson's linear and Spearman's rank correlation coefficients between different dominance indices based on 25 simulated quadrats, each quadrat having equal numbers of individuals and species.

	Simpson's dominance index ($\sum p_{i}^{2}$)	
Variances based indices	Pearson's Linear Correlation	Spearman's Rank Correlation
*Var*	1	1
$\frac{Var}{M^{2}}$	1	1
$\frac{Var}{M}$	1	1
$\frac{Var}{KM^{2}}$	1	1
$\frac{Var}{KM}$	1	1
$\frac{Var}{K}$	1	1
Standard deviation-based indices	Logarithmic function correlation	
*SD*	0.999	1
*CV*	0.999	1
*SE*	0.999	1

All correlations are significant at p < 0.001. K= Number of species, M = Mean number of individuals per species, Var = variance, SD = Standard deviation, CV = Coefficient of variation, SE = Standard error, *p* = probability of occurrence of a species.

**Fig. 6:**
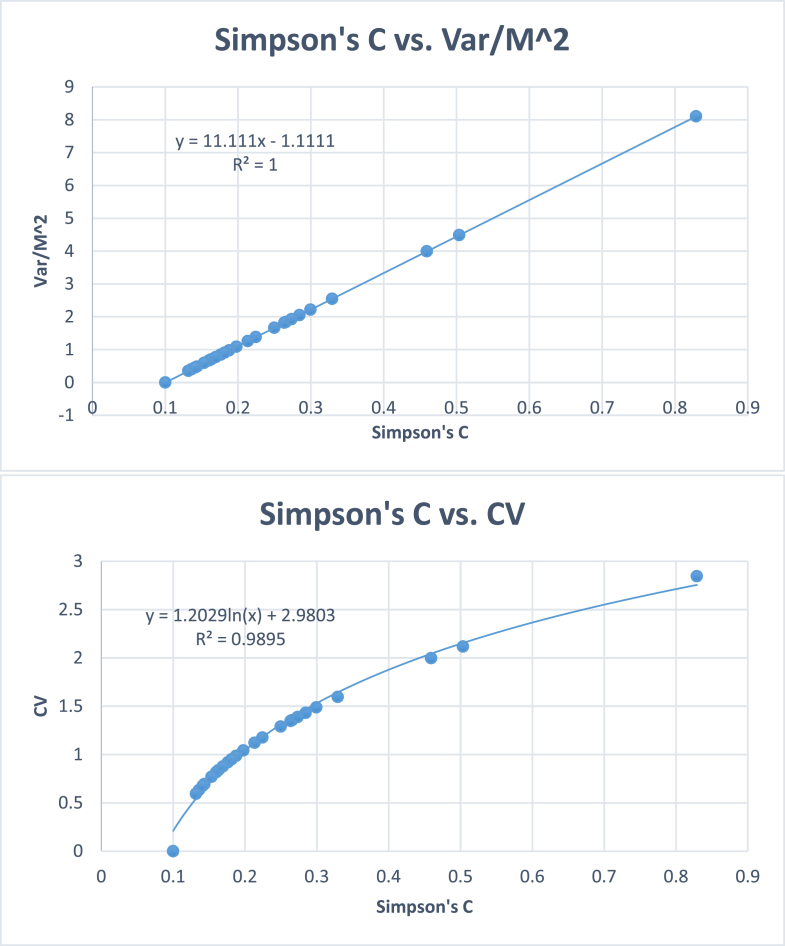
Plots between Simpson's dominance (C) using simulated data on 25 quadrats, each quadrat having 10 species and 100 individuals. Variance based indices give linear functions, whereas, standard deviation based indices give logarithmic functions.

**Fig. 7:**
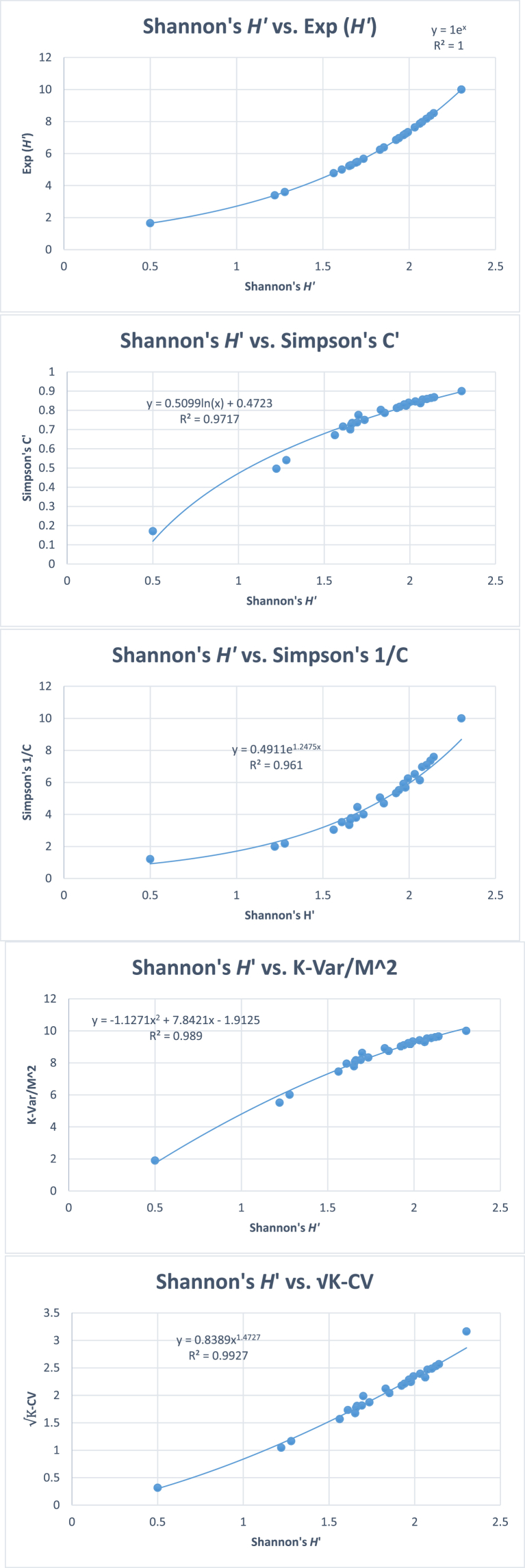
Plots between Shannon's *H′* and some other previous and new diversity indices using simulated data on 25 quadrats, each quadrat having 10 species and 100 individuals. All correlations are significant at p < 0.001.

**Fig. 8:**
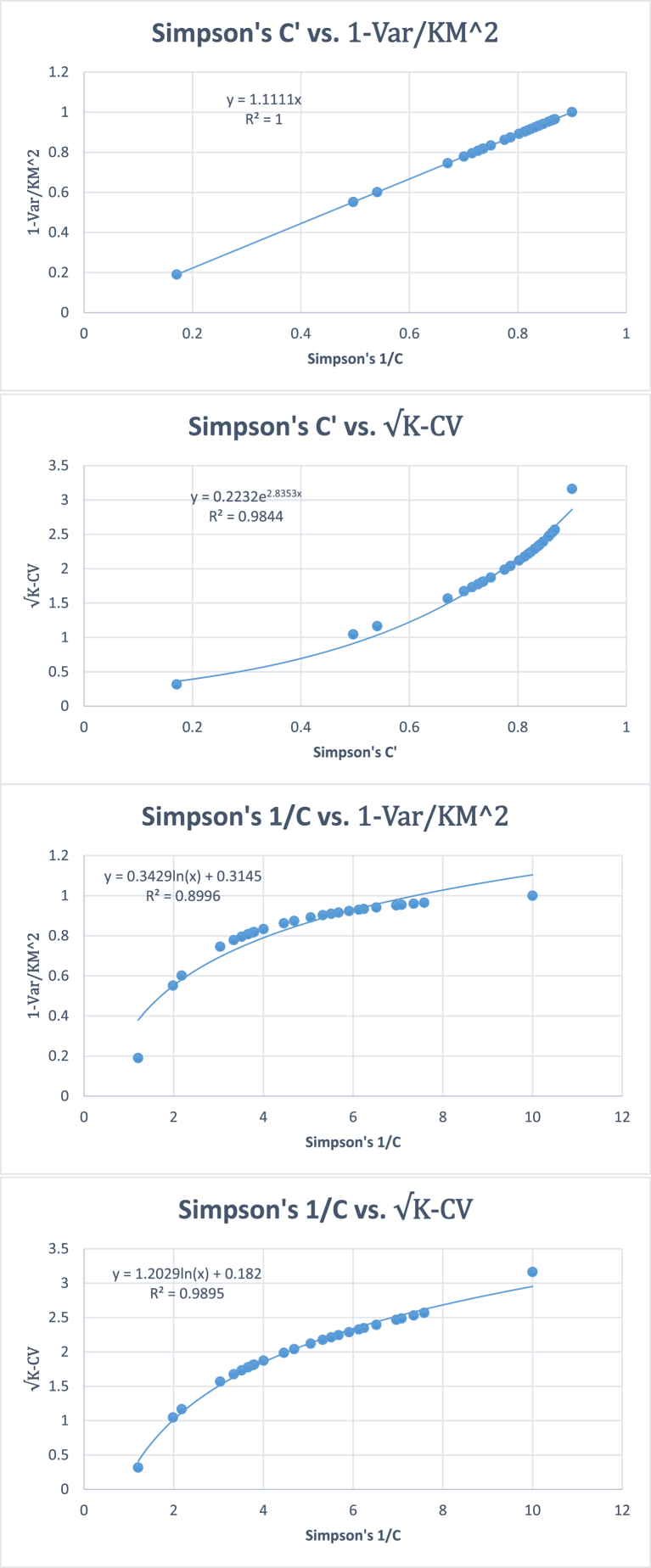
Plots between Simpson's indices of diversity with and some variance and standard deviation based proposed diversity indices using simulated data on 25 quadrats, each quadrat having 10 species and 100 individuals. All correlations are significant at p < 0.001.

However, dominance indices based on standard deviation were logarithmically correlated with Simpson's dominance with high degrees of correlation (Fig. 6). All the new indices have the same rank orders as that of Simpson's index. Similarly, diversity indices were also computed for the simulated data (Table 10, Figs. 7 and 8).

**Table 10:** Pearson's linear and Spearman's rank correlation coefficients between different diversity indices based on 25 simulated samples with equal numbers of individuals and species in each quadrat.

	*H*′	$1-\sum p_{i}^{2}$	$\frac{1}{\sum p_{i}^{2}}$	*H*′	$1-\sum p_{i}^{2}$	$\frac{1}{\sum p_{i}^{2}}$
	Pearson's Linear Correlation			Spearman's Rank Correlation		
*H*′		0.983	0.895		0.994	0.994
Exp (*H*′)	0.961	0.901	0.979	1	0.994	0.994
$1-\sum p_{i}^{2}$	0.983		0.819	0.994		1
$\frac{1}{\sum p_{i}^{2}}$	0.895	0.819		0.994	1	
Variance based diversity indices						
$M^{2}K-Var$	0.983	1	0.819	0.994	1	1
$K-\frac{Var}{M^{2}}$	0.983	1	0.819	0.994	1	1
$KM-\frac{Var}{M}$	0.983	1	0.819	0.994	1	1
$1-\frac{Var}{KM^{2}}$	0.983	1	0.819	0.994	1	1
$M-\frac{Var}{KM}$	0.983	1	0.819	0.994	1	1
$M^{2}-\frac{Var}{K}$	0.983	1	0.819	0.994	1	1
Standard deviation-based diversity indices						
$M\sqrt{K}-SD$	0.981	0.950	0.954	0.994	1	1
$\sqrt{K}-CV$	0.981	0.950	0.954	0.994	1	1
M−SE	0.981	0.950	0.954	0.994	1	1

All correlations are significant at p < 0.001. Better correlations can be obtained by using curvilinear regressions. K= Number of species, M = Mean number of individuals per species, Var = variance, SD = Standard deviation, CV = Coefficient of variation, SE = Standard error, p = probability of occurrence of a species.

Regarding diversity indices, all the indices gave significant positive linear correlations with Shannon's entropy and Simpson's index of diversity. Spearman's rank correlation analysis revealed that all the new variance and standard deviation-based indices follow the same rank orders as Simpson's index of diversity. However, the rank order correlations of the new diversity indices were slightly less than those of Simpson's index of diversity. Regressions between Shannon's and Simpson's diversity indices are not linear. Similarly, Shannon's index is nonlinearly related to the variance- and standard deviation-based diversity indices, and all these correlations are highly significant.

### 3.12 A case study of dominance and diversity analysis

New variance-based indices developed were calculated using plant abundance data from a community in the vicinity of the river Beas, in Punjab, India. The minimumquadrat size was 1 sq. m., and aminimum of five quadrats were required to sample the area. Table 11 gives the species composition of the site over 5 sq. m. area (5 quadrats X 1 sq. m.). The different indices proposed, along with the Simpson's and Shannon's indices calculated are given in Table 12.

**Table 11:** Number of individuals of different species from five quadrats of 1 sq. m. each from a site near river Beas, Punjab, India.

S. No.	Species	Number of individuals (*x*_*i*_)
1	*Ageratum conyzoides*L.	16
2	*Argemone mexicana*L.	4
3	*Cannabis sativa* L.	4
4	*Chenopodium ambrosioides* L.	16
5	*Erigeron bonariensis*L.	5
6	*Parthenium hysterophorus*L.	12
7	*Polygonum plebeium*R.Br.	7
8	*Ranunculussceleratus*L.	3
9	*Rumexdentatus*L.	5
Total	*K* = 9, *M* = 8	$\overset{9}{\underset{i=1}{\sum }}x_{i}=72$

**Table 12:** Dominance and diversity analysis of data from Table 11.

Dominance index	Dominance	Diversity index	Diversity	Diversity scale
*Var*	27.5	(*KM*^2^)-*Var*	548.5	0–576
*Var*/*M*^2^	0.43	*K*-(*Var*/*M*^2^)	8.57	0–9
*Var*/*M*	3.44	*KM*-(*Var*/*M*)	68.56	0–72
*Var*/*KM*^2^	0.05	1-(*Var*/*KM*^2^)	0.95	0–1
*Var*/*KM*	0.38	*M*-(*Var*/*KM*)	7.62	0–8
*Var*/*K*	3.1	M^2^ -(*Var*/*K*)	60.94	0–64
*SD*	5.24	(*M*√*K*)-*SD*	18.76	0–24
*CV*	0.66	√*K*-*CV*	2.34	0–3
*SE*	1.7	*M – SE*	6.25	0–8
Simpson's *C*, *C*′	0.15		0.85	
Shannon's *H′*			2.01	
Exp (*H′*)			7.52	

## 4 Discussion

Some of the commonly used diversity indices include Chao's method, Woodwell's Biodiversity index, Menhinick's index, Margalef's index, Odum's index, Berger-Parker's dominance index, Fisher's α, Brillouin's index, McIntosh's diversity index and Pielou's *J*, etc. [38]. The concept developed in this paper builds on the previous work conducted by the authors. Parkash and Thukral [26] proved that, similar to Shannon's and Simpson's indices, several statistics viz., geometric mean, harmonic mean, moments (μ_3_ and μ_4_), power mean, log mean, exponential mean and population variance (σ^2^) are also information measures and can be used as measures of dominance and diversity.

Sarangal et al. [39] gave a matrix method to develop Shannon's entropy, Simpson's dominance and some other information measures using diagonal and nondiagonal elements of a probability matrix. The binary probability plot for the commonly used Simpson's dominance index is convex, butis concave for Shannon's entropy [26, 38].

In the present study, the variance/mean square ratio diversity index and coefficient of variation diversity index present diversity on a scale of 0 to *K* species. This index is similar to the concept of the'number equivalent’ or the ‘effective number of species’, which is defined as the number of species that will give the same value as the diversity index [40]. In these terms, Shannon's Exp (*H′*) and Simpson's reciprocal diversity index are alsothe ‘effective number of species’. The maximum values of diversity indices having even distributions of species can be explained by Justus [41]. For communities having the same number of species, diversity is higher for communities representing species with equal numbers of individuals. It is seen that changes in samples with a lesser number of individuals will impact the index more. This can be explained in Whittaker [23], with an example from Simpson's index of dominance without replacement (C),

$$C\;\left(without\;replacement\right)=\overset{K}{\underset{i=1}{\sum }}\frac{n_{i}\left(n_{i}-1\right)}{N\left(N-1\right)},$$

where, *n*_*i*_ and *N* represent the number of individuals of *i*^*th*^ species, and thetotal number of individuals of all the species, respectively. If a quadrat consists of only a few individuals, any small change inthe number of individuals in the quadrat will affect the dominance and diversity indicesto a greatextent. The variance-to-mean square per species diversity index proposed by this paper will vary from 0 to 1 andis free from the sample size. It can also be used across diverse ecosystems. Other indices may also be used to quantify dominance and diversity depending upon the purpose of the analysis, i.e., the number of species, the mean number of individuals per species, or the total number of individuals in the quadrat or sample. In contrast, Shannon's index is only a diversity index and does not give the dominance of a community; instead, the new variance-based indices can be presented on a common dominance-diversity scale.

Correlation analysis revealed that the new variance and standard deviation-based dominance and diversity indices are significantly and positively correlated with probability-based Simpson's and Shannon's indices. Beck et al. [42] proved that one of the common drawbacks of measurement of β-diversity is under sampling, i.e., recording lesser numbers of the taxa than are actually present on the site. The authors coupled empirical data analysis with simulation studies and proved that this may lead to false conclusions. The present study on a α-diversity also needs to be tested empirically in order to work out appropriate sample sizes and the number of samples required for accurate phytosociological interpretations. A case study was undertaken to describe the procedure of deriving variance-based dominance and diversity indices (Tables 11 and 12). The present study proves that variance and standard deviation-baseddominance and diversity indices are complementary to each other and can be presented on a common scale.

If data on the number of individuals of different species is not available for the calculation of Shannon's and Simpson's indices, but data on descriptive statistics such as the number of species, the mean number of individuals and standard deviation is available, then the new indices may be useful.

## 5 Conclusions

**Fig. 9:**
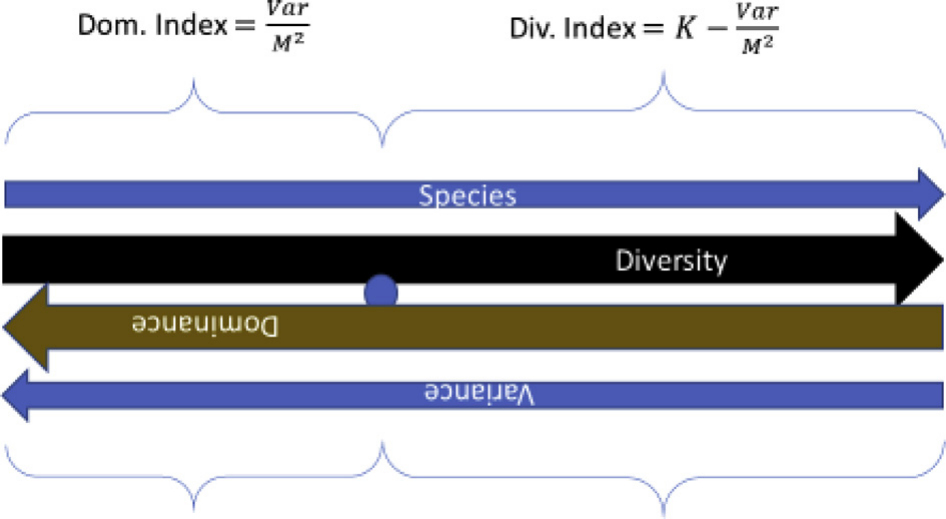
Relation between dominance and diversity with number of species (K) and variance.

This study described the calculation of Simpson's indices of dominance and diversity from the sample statistics. The study showed that variance and standard deviation of field data can be used effectively to describe the dominance and diversity in ecosystems. Rather than employing different indices to measure the dominance and diversity of a community on different scales, our hypothesis gives a method to measure these indices on a common scale (Fig. 9). If *K*is the number of species, *Var* is the variance, and *M* is the mean number of individuals per species in a quadrat, then Simpson's index of dominance may be determined from quadrat statistics,

$$\overset{K}{\underset{i=1}{\sum }}p_{i}^{2}=\left(\frac{K-1}{K^{2}}\right)\frac{Var}{M^{2}}+\frac{1}{K}\ldots \left(K\geq 1\right).$$

Gini-Simpson's index of diversity can be calculated accordingly as $\left(1-\overset{K}{\underset{i=1}{\sum }}p_{i}^{2}\right)$.Some of the new variance-based dominance and diversityindices proposed are:

**Table:** 

Index	Dominance index	Diversity index	Scale
Var.: mean sq. ratio	$\frac{Var}{M^{2}}dom.\;\;index$	$K-\frac{Var}{M^{2}}\;div.\;\;index$	0 – Spp.
Var. per sp: mean sq. ratio	$\frac{Var}{KM^{2}}\;dom.\;\;index$	$1-\frac{Var}{KM^{2}}\;div.\;\;index$	0–1
Coeff. variation	CVdom.index=CV	CVdiv.index=√K−CV	0 – √Spp.

We have also managed to define the variance (*S*^2^) and covariance (*Covar*) of a sample with only one value (*K* = 1) using the probability method.

$$\begin{array}{l} S^{2}=\frac{\sum_{i=1}^{K}{\left(x_{i}-M\right)}^{2}}{K-1}\ldots \left(K>1\right)\text{ ​}\\ \text{ ​ ​ ​ ​ ​}=M^{2}=x_{1}^{2}\ldots \left(K=1\right)\text{.} \end{array}$$

$$\begin{array}{l} Covar\left(X,Y\right)=\frac{\sum_{i=1}^{K}\left(x_{i}-M_{x}\right)\left(y_{i}-M_{y}\right)}{K-1}\ldots \left(K>1\right)\;\\ \;\;\;\;\;\;\;\;\;\;\;\;\;\;\;\;\;\;\;\;\;\;\;\;=M_{x}M_{y}=x_{1}y_{1}\ldots \left(K=1\right). \end{array}$$

This study will provide a new link between diversitystudies and statistics.

## Declarations

### Author contribution statement

Ashwani Kumar Thukral, Renu Bhardwaj: Conceived and designed the experiments; Wrote the paper.

Vinod Kumar: Performed the experiments; Wrote the paper.

Anket Sharma: Analyzed and interpreted the data; Wrote the paper.

### Funding statement

This research did not receive any specific grant from funding agencies in the public, commercial, or not-for-profit sectors.

### Competing interest statement

The authors declare no conflict of interest.

### Additional information

No additional information is available for this paper.
