# Corrigendum to “New indices regarding the dominance and diversity of communities, derived from sample variance and standard deviation” [Heliyon 5 (10) (October 2019) e02606]

**DOI:** 10.1016/j.heliyon.2019.e03017

**Published:** 2019-12-19

**Authors:** Ashwani Kumar Thukral, Renu Bhardwaj, Vinod Kumar, Anket Sharma

**Affiliations:** a Department of Botanical and Environmental Sciences, Guru Nanak Dev University, Amritsar, Punjab, 143005, India; b Department of Botany, DAV University, Sarmastpur, Jalandhar, Punjab, 144012, India; c State Key Laboratory of Subtropical Silviculture, Zhejiang A & F University, Hangzhou, 311300, China

In the original published version of this article, Table 5 incorrectly displayed the term “Correl.(X,Y)” in column 1, row 12. This was an error and should have read “Covar/(M_X_M_Y_)”. The authors apologise for this error, which does not affect the results, conclusions, or discussion in the article. The corrected table is available below. Both the HTML and PDF versions of the article have been updated to correct the error.

**Table. 5:** Sample covariance between pairs of single value variables, *X* and *Y*, with one of the values of *x*_*i*_>0, and the other values of *x* equal to zeroes.

Sample size, (number of species, *K*)	*K*=2		*K*=3		*K*=4		*K*=5		*K*=6	
Samples (*X,Y*)	*X*	*Y*	*X*	*Y*	*X*	*Y*	*X*	*Y*	*X*	*Y*
Species	Number of individuals									
*x*_1_ >0	10	4	10	4	10	4	10	4	10	4
*x*_2_	0	0	0	0	0	0	0	0	0	0
*x*_3_			0	0	0	0	0	0	0	0
*x*_4_					0	0	0	0	0	0
*x*_5_							0	0	0	0
*x*_6_									0	0
Mean (*M*)	5	2	3.33	1.33	2.5	1	2	0.8	1.66	0.66
Covar.(X,Y)	20		13.33		10		8		6.66	
$Covar/\left(M_{x}M_{y}\right)$	2		3		4		5		6	
Regr.(X,Y)	0.4		0.4		0.4		0.4		0.4	

The values 10 and 4 are tentative. Any set of values of *x*_1_> 0 will give the *Covar*/(*M*_1_*M*_2_) ratio equal to *K*.

